# Progranulin Administration Attenuates β-Amyloid Deposition in the Hippocampus of 5xFAD Mice Through Modulating BACE1 Expression and Microglial Phagocytosis

**DOI:** 10.3389/fncel.2020.00260

**Published:** 2020-08-18

**Authors:** Zhangxin Guan, Zuolong Chen, Shumei Fu, Linbin Dai, Yong Shen

**Affiliations:** ^1^Institute on Aging and Brain Disorders, The First Affiliated Hospital of USTC, Division of Life Sciences and Medicine, University of Science and Technology of China, Hefei, China; ^2^Hefei National Laboratory for Physical Sciences at the Microscale, Neurodegenerative Disorder Research Center, School of Life Sciences, University of Science and Technology of China, Hefei, China; ^3^Center for Excellence in Brain Science and Intelligence Technology, Chinese Academy of Sciences, Shanghai, China

**Keywords:** Alzheimer’s disease, progranulin, Aβ, BACE1, microglia

## Abstract

Loss of function mutations in the progranulin (PGRN) gene is a risk factor for Alzheimer’s disease (AD). Previous works reported that the deficiency of PGRN accelerates β-amyloid (Aβ) accumulation in AD transgenic mouse brains while overexpression of PGRN could restrain disease progression. However, mechanisms of PGRN in protecting against Aβ deposition remains unclear. Here, using the 5xFAD AD mouse model, we show that intrahippocampal injection of PGRN protein leads to a reduction of Aβ plaques, downregulation of beta-secretase 1 (BACE1), and enhanced microglia Aβ phagocytosis in the mouse hippocampus. Furthermore, PGRN treatment inhibited BACE1 expression in N2a cells and primary culture neurons and improved the phagocytic capacity of microglia isolated from 5xFAD mouse brains. Collectively, our results provide further evidence that enhancing progranulin could be a promising option for AD therapy.

## Introduction

Alzheimer’s disease (AD) is an irreversible neurodegenerative disorder which is characterized by progressive impairment of learning and memory. Extracellular accumulation of amyloid-β (Aβ) plaques and intracellular neurofibrillary tangles (NFTs) are two well recognized pathological features of AD (Hardy and Higgins, 1992). AD is the most common cause of dementia among the elderly (Panegyres, 2004). As most of the AD onset is over the age of 65, aging is considered to be a major risk factor for AD (Xia et al., 2018).

In the AD brain, the imbalance between Aβ generation and clearance is an early event that leads to long-term accumulation and aggregation of Aβ, which finally results in amyloid plaque deposition (Cavallucci et al., 2012). Aβ is produced from sequential proteolytic cleavage of amyloid precursor protein (APP) by β-secretase (BACE1) and γ-secretase. As the rate-limiting enzyme in the formation of Aβ, BACE1 is one of the most potential therapeutic targets for AD (Cai et al., 2001). On the other hand, the clearance of Aβ is accomplished by several mechanisms including microglial phagocytosis. Microglia are “professional” phagocytes in the central nervous system and play a dominant role in aggregated protein clearance. Thus, enhancing microglial phagocytosis also be proposed as a potential therapeutic strategy for AD (Lee and Landreth, 2010; Yoon and Jo, 2012; Maleki and Rivest, 2019).

Progranulin (PGRN) is a pleiotropic protein involved in many biological processes. In the brain, PGRN is mainly expressed in microglia and neurons and plays a beneficial role in the regulation of neuroinflammation and neuronal survival (Van Damme et al., 2008; Martens et al., 2012). The mutation in PGRN is regarded as a risk factor for AD as well as frontotemporal lobar degeneration (FTLD; Brouwers et al., 2008; Perry et al., 2013). In the AD brain, PGRN is colocalized with Aβ plaques (Petkau and Leavit, 2014). Previous studies have demonstrated that PGRN deficiency promotes Aβ deposition, and overexpression of PGRN reduced Aβ plaque burden in several AD transgenic mouse models (Minami et al., 2014; Van Kampen and Kay, 2017). However, the underlying mechanisms of PGRN in protecting from Aβ deposition remain largely unclear.

In this study, to explore the direct effect of PGRN on Aβ pathology, we injected recombinant PGRN protein into the brain of 5xFAD mice, a commonly used AD mouse model (Mazi et al., 2018). Our results demonstrate that Aβ deposition in the hippocampus of 5xFAD mice was significantly reduced after PGRN administration. Furthermore, BACE1 expression and microglial phagocytosis were modulated by PGRN. Consequently, our data indicated that PGRN attenuates Aβ pathology in 5xFAD mice through multiple pathways.

## Materials and Methods

### Mice

5xFAD mice were originally purchased from the Jackson Laboratory (Bar Harbor, ME, USA). All mice were bred and fed ad libitum and maintained a regular light-dark cycle of 12 h. Mice of both genders were used for experiments. All animal experiments were reviewed and approved by the Institutional Animal Care and Use Committee of the University of Science and Technology of China.

For genotyping of 5xFAD mice by PCR, the following primer pairs were used: forward primer (5′-AGG ACT GAC CAC TCG ACC AG-3′) and reverse primer (5′-CGG GGG TCT AGT TCT GCA T-3′).

### Stereotaxic Injections

The recombinant mouse PGRN protein solution was prepared according to the instructions. PGRN powder (2557-PG-050, R&D) was dissolved in sterile PBS as a stock solution (250 μg/ml) and store at 4°C within 1-month, or −20°C for long-term storage. 5xFAD mice at 4 months old were anesthetized with 2% sodium pentobarbital (20 μl/g body weight) and secured on a stereotaxic frame (RWD, Shenzhen, China). Two microlitre PBS was delivered to the hippocampus of the left hemisphere and 2 μl PGRN solution was delivered to the hippocampus of right hemisphere at a rate of 150 nl min^−1^ [anteroposterior (AP) −1.9, mediolateral (ML) ± 2.0, dorsoventral (DV) −2.6]. Mice were sacrificed after 3 weeks and the brains were harvested.

### Brain Harvest and Immunofluorescence

Animals were anesthetized and perfused using PBS, and brains were fixed in 4% paraformaldehyde (PFA) for 24 h, then incubated in 15% and 30% sucrose for cryoprotection. Sections (30 μm) of the brain were cut using a Leica CM3050 S freezing microtome, permeabilized with 0.3% Triton X-100 in PBS buffer, and blocked with 5% normal goat serum for 1 h. Sections were incubated with anti-Iba1 (019-19741, 1:500, Wako, Osaka, Japan), PE anti-CD68 (137013, 1:100, Biolegend), anti-TREM2 (sc-373828, 1:200, Santa), 4G8 (800702, 1:500, Biolegend), or anti-BACE1 (5606S, 1:500, CST) antibodies overnight at 4°C, followed by incubation with anti-mouse Alexa Fluor^®^488 Conjugate (4408S, 1:500, CST) or anti-rabbit Alexa Fluor^®^555 Conjugate (4413S, 1:500, CST) secondary antibodies for 1 h at room temperature. For Thioflavin S (T1892, Sigma) staining, sections were stained with 0.015% Thio-S in 50% ethanol for 8 min at room temperature.

Images were acquired using a TissueGnostics TissueFAXS system or Zeiss LSM800 confocal microscopy. The area and number of Aβ plaques in the hippocampus were analyzed using ImageJ. Three brain sections per hemisphere (about 300 μm apart) were analyzed, and the average percentages of the hippocampal area occupied by plaques were calculated. The total fluorescence intensity of BACE1 in the hippocampus was analyzed using ImageJ software. All values were normalized to the left hemisphere (PBS) levels for each mouse.

### Quantitative Real-Time PCR

To examine the transcriptional changes of BACE1 following PGRN treatment, N2a cell lines were plated in a 12-well culture plate, and different concentrations of PGRN were added to wells. After overnight incubation, total RNAs were isolated using TRIzol^®^ Reagent (15596018, Invitrogen), then reversely transcribed to cDNA using the PrimeScript^®^ RT reagent kit (RR047A, TaKaRa). qPCR reactions were performed using TransStart^®^ Tip Green qPCR SuperMix (AQ141, Transgen, Beijing, China). The expression of GAPDH was used as an endogenous control. Primer sequences were as follows: BACE1 (Jiang et al., 2014), forward 5′-CAG TGG AAG GTC CGT TTG TT-3′, reverse 5′- CTA AAG GAT GCT GGG CAG AG-3′; GAPDH, forward 5′-CCC TTC ATT GAC CTC AAC TA-3′, reverse 5′-CCT TCT CCA TGG TGG TGA A-3′.

Experiments were repeated four times. Relative expression levels of BACE1 were calculated using the 2^−ΔΔCT^ method and normalized to GAPDH.

### Western Blot

For N2a cells, cells were plated in a 12-well culture plate, and different concentrations of PGRN were added to wells. After overnight incubation, total cell extracts were lysed with 100 μl radioimmunoprecipitation assay (RIPA) buffer containing protease inhibitors (4693116001, Roche). Nuclear and Cytoplasmic Protein Extraction Kit (P0027, Beyotime) was used for nuclear protein and cytoplasmic protein extraction.

For primary neuronal cultures, cortex and hippocampus from postnatal day 1 (P1) mouse pups were chopped and minced in DMEM medium (SH30022. 01, HyClone) with 20 U/ml papain (LS003127, Worthington). After 20 min incubation at 37°C, the enzyme was deactivated with medium containing 20% FBS and passing through a 70-μm filter. After washing, isolated cells were resuspended in DMEM medium containing 10% FBS, penicillin (100 U/ml) and streptomycin (100 μg/ml), then were plated into 12-well culture plates coated with poly-D-lysine. After culturing for 4 h, the medium was replaced with neurobasal medium (21103049, Thermofisher) supplemented with B27. After culture for 7 days, different concentrations of PGRN were added to wells and incubated for another 24 h, then total cell extracts were lysed with 100 μl RIPA buffer.

Proteins were loaded onto either 10% or 17% SDS-PAGE gel and transferred to a PVDF Blotting membrane, blocked with 5% nonfat dry milk and probed for APP, NF-κB p-IκBα, IκBα and BACE1 with anti-APP (A8717, 1:1,000, Sigma), anti-NF-κB (8242, 1:1,000, CST), anti-p-IκBα (9246, 1:1,000, CST), anti-IκBα (4814, 1:1,000, CST) and anti-BACE1 (5606S, 1:1,000, CST) antibodies respectively. GAPDH was used as an internal reference. After washing, membranes were incubated with HRP-conjugated anti-mouse IgG (H ± L; W4011, 1:4,000, Promega) or HRP-conjugated anti-rabbit IgG (H ± L; W4021, 1:4,000, Promega), and antibody binding was detected using SuperSignal™ West Femto Maximum Sensitivity Substrate (34096, Thermo Scientific).

### Phagocytosis Assay

Adult microglia were isolated and cultured as previously described (Singh et al., 2014). Briefly, brains of 5xFAD mice at 2 months of age were perfused with 1xPBS, chopped and minced in DMEM medium (SH30022. 01, HyClone) with 20 U/ml papain (LS003127, Worthington). After 25 min incubation at 37°C, enzymes were deactivated with an equal volume of DMEM medium containing 20% FBS. After passing through a 70-μm filter, cells were centrifuged at 1,000 g for 5 min at 4°C and resuspended in 30% Percoll (17-0891-09, GE Healthcare). After centrifuged at 500 g for 20 min, the 30% Percoll layer was removed carefully and the cell pellet was collected for further processing.

Isolated microglia were plated into 24-well cell imaging plates (around 2 × 10^5^) coated with poly-D-lysine, and cultured in an incubator at 37°C, 5% CO_2_. To examine the effect of microglial phagocytosis by PGRN, seeded microglia were pre-incubated with fluorescent latex beads of 1 μm in diameter (L1030, 1:400 Sigma-Aldrich) in DMEM medium (SH30022. 01, HyClone) for 1 h, and an equal volume of DMEM medium containing different concentrations of PGRN (0 and 500 ng/ml) was added for another 1 h. Treated cells were fixed with 4% PFA for 15 min, then permeabilized with 0.3% Triton X-100 in PBS buffer for 5 min and blocked with 5% normal goat serum for 30 min. Cells were incubated with anti-Iba1 (019-19741, 1:500, Wako) antibody at 4°C overnight, then incubated in anti-rabbit Alexa Fluor^®^555 Conjugate secondary antibody (4413S, 1:500, CST) and DAPI (D1306, 1:1,000, Invitrogen) for 15 min at RT. Images were acquired using the Zeiss LSM800 confocal microscopy system. Quantitative analyses of Iba1 and fluorescent latex beads areas were performed with ImageJ.

### Statistical Analyses

Data were analyzed with GraphPad Prism 5 and expressed as mean ± SD, the significance of differences was evaluated with the student’s paired *t*-test or student’s unpaired *t*-test. *P* < 0.05 was considered significant.

## Results

### Administration of PGRN in the Hippocampus of 5xFAD Mice Attenuates Aβ Deposition

**Figure 1:**
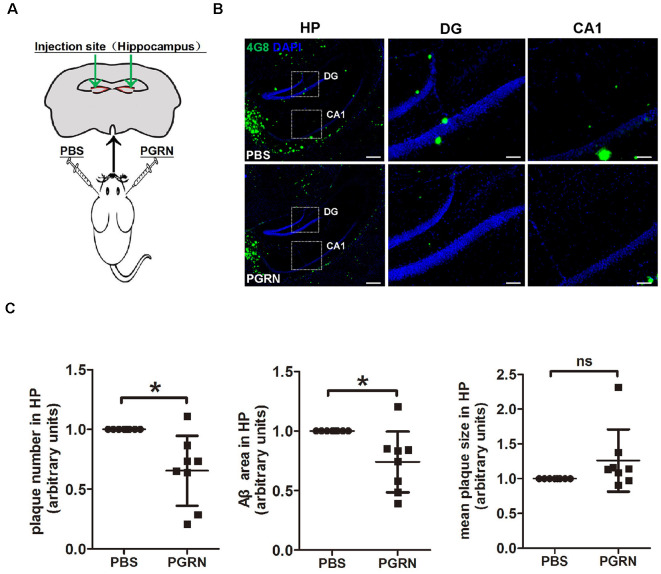
Progranulin (PGRN) administration attenuates β-amyloid (Aβ) deposition in the hippocampus of 5xFAD mice. **(A)** Schematic diagrams of PBS or PGRN injection into the mice hippocampus. The right hemisphere was injected with PGRN and the left hemisphere was injected with PBS as self-control. **(B)** Representative images of Aβ plaques of the hippocampus (HP) of 5xFAD mice injected with PBS (control) or PGRN. Aβ (4G8, green) and DAPI (blue). Scale bars, 200 μm (HP), 50 μm (DG, CA1). **(C)** Quantification of the area, number and mean size of Aβ plaques in the hippocampus, all values were normalized to left hemisphere levels for each mouse, *n* = 8, *p < 0.01, paired student’s *t*-test. ns, not significant.

In an attempt to check whether direct PGRN supplement could influence AD pathology, we injected recombinant mouse PGRN protein into 4-month-old 5xFAD mouse hippocampus, a region of the brain that essential for learning and memory. To exclude the influence of individual differences, PBS was delivered to the left hemisphere as control, and PGRN was delivered to the right hemisphere in the same animal (Figure 1A). After 3 weeks, the injected mice were sacrificed and brain tissues were collected. Immunofluorescence staining of Aβ plaques with 4G8 antibody showed that the levels Aβ deposition in the hippocampus were significantly lower in the side injected with PGRN than in control side (Figure 1B), as quantified by the total area and number of plaques, although there was no difference in mean plaques size (Figure 1C). This suggested that direct PGRN delivery could alleviate Aβ pathology.

### PGRN Inhibits BACE1 Expression

Aβ is a peptide derived from APP processing. During this process, BACE1 is the key enzyme and its expression levels were significantly correlated with Aβ pathology (Rossner et al., 2006). BACE1 expression level is increased in the brain of AD patients (Cheng et al., 2014). It is believed that chronic inflammatory response, which is involved in the pathological process of AD, promotes BACE1 expression (Wen et al., 2004; Sastre et al., 2006). Given that PGRN has anti-inflammatory properties (Wu and Siegel, 2011; Lee et al., 2017), we naturally sought to determine whether PGRN could regulate BACE1 expression.

**Figure 2:**
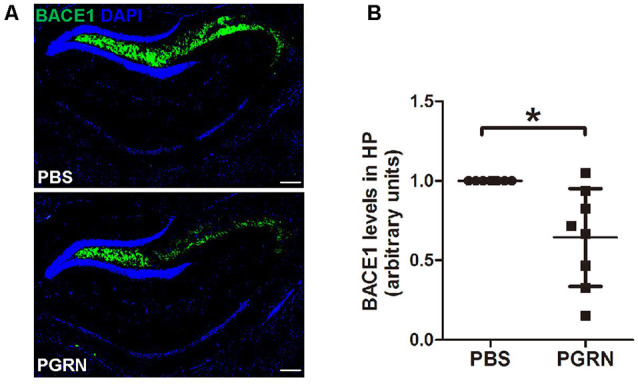
PGRN administration reduces β-secretase (BACE1) levels in the hippocampus of 5xFAD mice. **(A)** Representative images of BACE1 of the hippocampus of 5xFAD mice injected with PBS (control) or PGRN. BACE1 (green) and DAPI (blue). Scale bars, 200 μm. **(B)** Quantification of BACE1 immunostaining in the hippocampus, all values were normalized to left hemisphere levels for each mouse, *n* = 8, *p < 0.05, paired student’s *t*-test.

First, we examined whether BACE1 expression levels were altered in the hippocampus after PGRN injection. Interestingly, immunofluorescence staining showed that BACE1 protein levels were lower in the side of brain sections with PGRN injection compared to the control side, as quantified by the total fluorescence intensity (Figures 2A,B).

**Figure 3:**
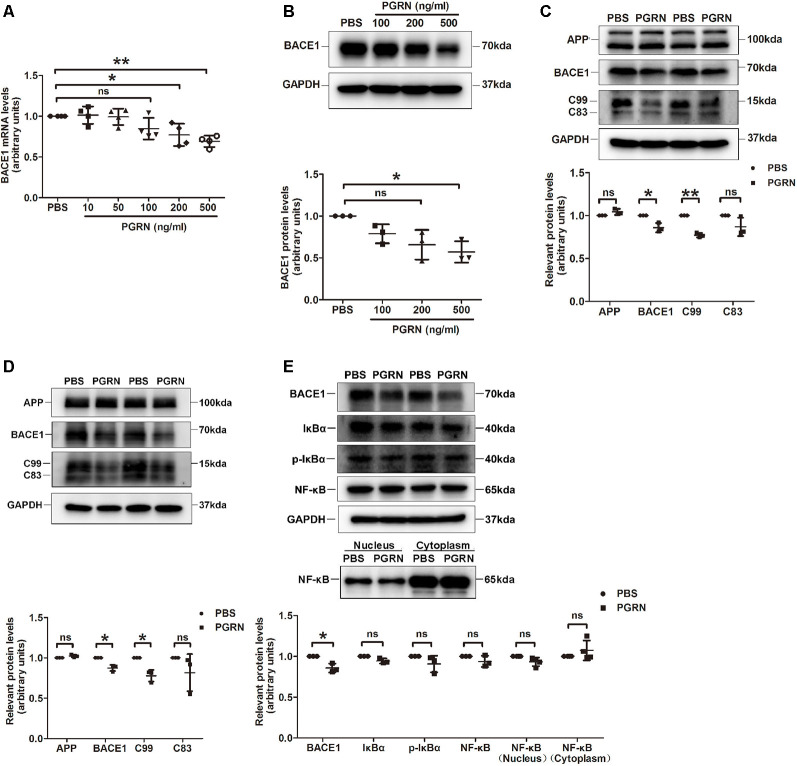
PGRN treatment inhibits BACE1 expression. **(A)** BACE1 expression was determined by qRT-PCR in N2a cells after treated with different concentration PGRN, all values were normalized to the group treated with PBS for each experiment. Data were from four independent experiments, *p < 0.05, **p < 0.01, paired student’s *t*-test. **(B)** Levels of BACE1 in N2a cells treated with different concentration PGRN were determined by western blot, all values were normalized to the group treated with PBS for each experiment. *n* = 3 independent experiments, *p < 0.05, paired student’s *t*-test. **(C)** Levels of amyloid precursor protein (APP), BACE1, C99 and C83 in N2a cells treated with 500 ng/ml PGRN were determined by western blot, all values were normalized to the group treated with PBS levels for each experiment. Data were from three independent experiments, *p < 0.05, **p < 0.01, paired student’s *t*-test. **(D)** Protein levels of amyloid precursor protein (APP), BACE1, C99 and C83 in primary culture neurons treated with 500 ng/ml PGRN were determined by western blot, all values were normalized to the group treated with PBS levels for each experiment. Data are from three independent experiments, *p < 0.05, paired student’s *t*-test. **(E)** Protein levels of BACE1, IκB, p-IκB, and NF-κB in N2a cells treated with 500 ng/ml PGRN were determined by western blot. All values were normalized to the group treated with PBS levels for each experiment. Data were from three independent experiments, *p < 0.05, paired student’s *t*-test. ns, not significant.

To further determine whether PGRN could inhibit BACE1 expression, we treated N2a cells, a mouse neuroblastoma cell line with high BACE1 expression, with various concentrations of PGRN *in vitro*. BACE1 mRNA levels were gradually reduced with the increase of PGRN concentration and reached significance at 200 ng/ml (Figure 3A). Consistently, BACE1 protein levels detected by western blot were also decreased after PGRN treatment (Figure 3B). We also checked the C-terminal fragments (CTFs) produced during APP processing. C99, which is generated by BACE1 cleavage of APP, was significantly decreased after PGRN treatment. In contrast, C83, which is generated in the non-amyloid pathway by α-secretase cleavage of APP, showed no significant difference after PGRN treatment (Figure 3C). The same results were found in PGRN treated primary culture neurons (Figure 3D). Taken together, PGRN reduced Aβ accumulation in the brain of 5xFAD mice was probably by inhibiting BACE1 expression.

Next, we investigated the mechanism of how PGRN inhibits BACE1 expression. It has been reported that PGRN can attenuate NF-κB activation mediated by pro-inflammatory cytokines (Hwang et al., 2013), while NF-κB signaling facilitates BACE1 expression by directly binding to BACE1 promoter (Chen et al., 2012; Wang et al., 2013). Therefore, we asked whether PRGN exerts its effect on BACE1 expression by targeting the NF-κB signaling pathway. Unfortunately, we didn’t find a significant difference in NF-κB activation, although PGRN treatment showed a tendency of reducing NF-κB translocation from the cytoplasm to the nucleus (Figure 3E).

### PGRN Enhances Microglial Phagocytosis

**Figure 4:**
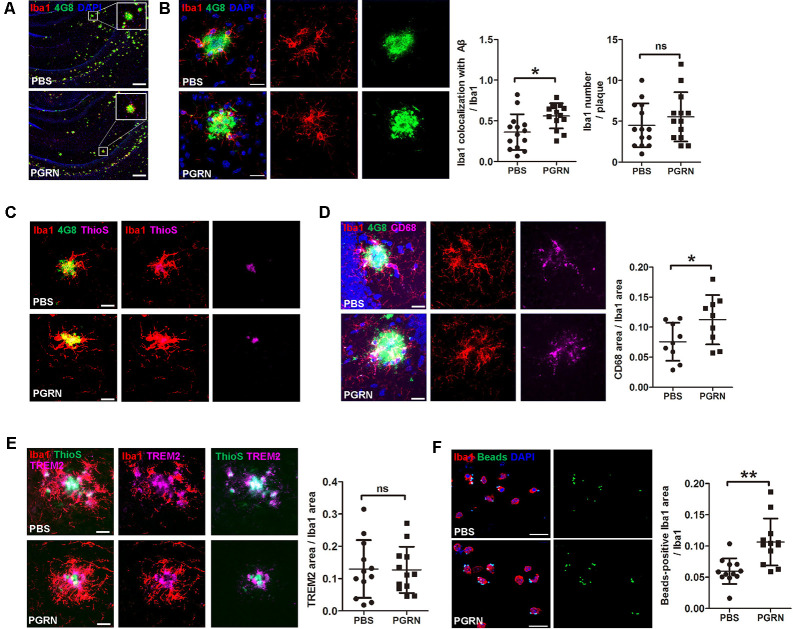
PGRN enhances microglial phagocytosis. **(A)** Representative images of microglia with Aβ in the hippocampus of 5xFAD mice injected with PBS (control) or PGRN. microglia (Iba1, red), Aβ (4G8, green), and DAPI (blue). Scale bars, 200 μm. **(B)** Representative images and quantification of colocalization of microglia with Aβ. microglia (Iba1, red), Aβ (4G8, green), and DAPI (blue). Scale bars, 20 μm, *n* = 14 from three mice, *p < 0.05, unpaired student’s *t*-test. **(C)** Representative images of microglia with Aβ. Aβ (4G8, green), Aβ (ThioS, purple), and microglia (Iba1, red). Scale bars, 20 μm. **(D)** Representative images of microglia with Aβ and quantification of CD68-positive Iba1 area. Aβ (4G8, green), microglia (Iba1, red), microglia (CD68, purple), and DAPI (blue). Scale bars, 20 μm. *n* = 9 from three mice, *p < 0.05, unpaired student’s *t*-test. **(E)** Representative images of microglia with Aβ and quantification of the TREM2-positive Iba1 area. Aβ (ThioS, green), microglia (Iba1, red), and TREM2 (purple). Scale bars, 20 μm, *n* = 12 from three mice, *p < 0.05, unpaired student’s *t*-test. **(F)** Representative images and quantification of fluorescent beads phagocytosis assay using isolated microglia from adult 5xFAD mouse brain and treated with PBS (control) or 500 ng/ml PGRN. microglia (Iba1, red), beads (green), and DAPI (blue). Scale bars, 20 μm, *n* = 12 fields of view from three independent experiments, **p < 0.01, unpaired student’s *t*-test. ns, not significant.

A previous study reported that microglial PGRN deficiency leads to impaired phagocytosis (Minami et al., 2014). Given the essential role of microglial phagocytosis in the clearance of Aβ (Mosher and Wyss-Coray, 2014), we next investigated whether PGRN supplement could enhance microglial phagocytosis in the brain of 5xFAD mice. Mouse brain frozen sections were stained with anti-Iba1 and anti-Aβ antibodies (4G8) to co-label microglia and Aβ plaques. The results showed that microglia clustered much adjacent to the center of Aβ plaques after PGRN injection, whereas activated microglia mainly distributed around the Aβ plaques in the control group (Figures 4A,B). Consistently, double staining of Thioflavine S (a fluorescent histochemical marker of dense core Aβ plaques) with Iba1 also showed that microglia was closer to plaque core in PGRN-injected group (Figure 4C). Although no significance in microglial recruitment was observed as determined by quantitative analysis of the number of microglia surrounding the plaques, the colocalization of microglia with Aβ significantly increased (Figure 4B). Furthermore, CD68, a marker for phagocytosis, was increased in Iba1^+^ microglia in close contact with Aβ plaques (Figure 4D). However, PGRN had no significant effect on TREM2 (triggering receptor expressed on myeloid cells 2) expression, a marker of stage 2 disease-associated microglia (DAM; Keren-Shaul et al., 2017; Figure 4E).

To further demonstrate that PGRN could promote microglial phagocytosis, we isolated microglia from the brain of adult 5xFAD mice for primary culture and incubated with PGRN *in vitro* to perform a phagocytosis assay. As expected, PGRN treated microglia phagocytosed more fluorescent latex beads than PBS-treated groups (Figure 4F). Taken together, these data suggest that PGRN promotes microglial phagocytosis to facilitate the clearance of Aβ in the brain of 5xFAD mice.

## Discussion

Here we show that administration of PGRN attenuates Aβ deposition in the hippocampus of 5xFAD mice, with decreased BACE1 levels and enhanced microglial phagocytosis. Moreover, PGRN treatment reduced BACE1 expression of cultured N2a cells and primary culture neurons *in vitro* and promoted phagocytosis of microglia isolated from adult 5xFAD mice. Thus, PGRN may play an essential role in AD pathology through modulating the production and clearance of Aβ, which further supports the proposal that PGRN-enhancing therapy could be a hopeful strategy for AD treatment.

Moreover, our data reveal a potentially novel role of PGRN in inhibiting BACE1 expression. Many factors such as hypoxia, oxidative stress, and inflammation can increase BACE1 expression (Rossner et al., 2006; Sun et al., 2012), PGRN is widely recognized as a multi-functional protein such as against oxidative stresses, anti-apoptotic and anti-inflammatory properties. It has been shown that PGRN could bind directly to TNFR to inhibit NF-κB activation (Tang et al., 2011). Indeed, our previous studies have elucidated a critical role of the TNFα/TNFR signaling pathway in AD pathology through regulating BACE1 expression, which mediated by the NF-κB pathway (He et al., 2007; Jiang et al., 2014). Overexpression of NF-κB was able to promote BACE1 expression (Bourne et al., 2007; Chen et al., 2012). In our study, we found no obvious difference in NF-κB activation upon PGRN treatment. Thus, NF-κB may dispensable for BACE1 upregulation under this condition. Of note, Other factors which control BACE1 expression including JAK/STAT pathway, PPARγ and specificity protein 1 (SP1), had also been reported that be regulated by PGRN (Sun et al., 2012; Yang et al., 2013; Yan et al., 2016; Li et al., 2018). Given these pieces of evidence, we believe that PGRN probably regulates the expression of BACE1 through another target. Further studies are needed to reveal the exact mechanisms of how PGRN interferes with BACE1 expression.

Microglia are brain resident immune cells that play crucial roles in the immune surveillance of the central nervous system (Norris and Kipnis, 2019). Microglia can either exert neurotoxic or neuroprotective function in AD pathogenesis, through the production of proinflammatory cytokines or phagocytosis of aggregated Aβ proteins respectively. These distinct outcomes largely depend on microglial phenotypes and disease stage (Sarlus and Heneka, 2017; Zhou et al., 2018). One feature of microglia in AD pathology is that they cluster around plaques for Aβ clearance. It has been reported that PGRN can recruit microglia to phagocytose Aβ (Pickford et al., 2011). Moreover, PGRN deficiency impairs phagocytosis of microglia and increases Aβ deposition (Minami et al., 2014). Consistently, we found that PGRN enhanced the colocalization of microglia with Aβ and increased CD68 expression in Iba1^+^ microglia *in vivo*, and promoted endocytosis capacity of microglia isolated from adult 5xFAD mouse brain. TREM2 was highly expressed in microglia around plaques both in control and PGRN treated groups, which indicate DAM phenotypes. However, despite our and other groups reports, mechanisms of how PGRN enhances microglial phagocytosis remains poorly understood. It is well known that the phagocytic capacity of phagocytes largely relies on lysosome function. Growing evidence shows that PGRN plays a key role in regulating lysosome biogenesis and function (Kao et al., 2017), it is likely that PGRN affects microglial phagocytosis through regulating lysosome function. In support of this idea, a recent striking study showed that in *Grn*^−/−^ mice, microglia accumulate high numbers of lipid droplets, resulting in lysosomal dysfunction and phagocytosis deficits (Marschallinger et al., 2020).

There are conflicting results in previous studies regarding the consequences of PGRN deficiency on AD pathology (Minami et al., 2014; Takahashi et al., 2017; Van Kampen and Kay, 2017). The debate may attribute to different forms of Aβ during disease progress that has different effects on PGRN expression. In the early stage of AD, PGRN levels are reduced, while in older mice with extensive plaque pathology or advanced AD patients, PGRN levels are increased (Minami et al., 2014; Satoh et al., 2014). On the other hand, considering the multiple roles of PGRN, it is probable that PGRN has divergent functions at different disease stages. How PGRN is regulated and the exact features of PGRN at a different stage of AD are currently unclear.

Overall, our data suggest that PGRN supplementation protects against Aβ deposition in 5xFAD mice at least in two ways, one is reducing the production of Aβ through inhibiting BACE1 expression, and another is promoting the clearance of Aβ *via* enhancing microglial phagocytosis. In future studies, it will be crucial to determine whether and how PGRN affects the function of brain cells besides neuron and microglia during the process of aging and AD.

## Data Availability Statement

All datasets generated for this study are included in the article.

## Ethics Statement

The animal study was reviewed and approved by the Institutional Animal Care and Use Committee of the University of Science and Technology of China.

## Author Contributions

ZG and ZC performed experiments, acquired the data, and drafted the manuscript. LD and SF prepared biological reagents. ZC and YS assisted in experimental design and data analysis, revised the manuscript, and provided funding.

## Conflict of Interest

The authors declare that the research was conducted in the absence of any commercial or financial relationships that could be construed as a potential conflict of interest.
